# In Silico Studies of Some Isoflavonoids as Potential Candidates against COVID-19 Targeting Human ACE2 (hACE2) and Viral Main Protease (M^pro^)

**DOI:** 10.3390/molecules26092806

**Published:** 2021-05-10

**Authors:** Mohamed S. Alesawy, Abdallah E. Abdallah, Mohammed S. Taghour, Eslam B. Elkaeed, Ibrahim H. Eissa, Ahmed M. Metwaly

**Affiliations:** 1 Medicinal Pharmaceutical Chemistry & Drug Design Department, Faculty of Pharmacy (Boys), Al-Azhar University, Cairo 11884, Egypt; Abdulla_emara@azhar.edu.eg (A.E.A.); Mohammad1533.el@azhar.edu.eg (M.S.T.); 2 Department of Pharmaceutical Sciences, College of Pharmacy, AlMaarefa University, Ad Diriyah, Riyadh 13713, Saudi Arabia; ikaeed@mcst.edu.sa; 3 Department of Pharmaceutical Organic Chemistry, Faculty of Pharmacy (Boys), Al-Azhar University, Cairo 11884, Egypt; 4 Department of Pharmacognosy and Medicinal Plants, Faculty of Pharmacy (Boys), Al-Azhar University, Cairo 11884, Egypt; ametwaly@azhar.edu.eg

**Keywords:** COVID-19, isoflavonoids, molecular docking, human ACE2, main protease

## Abstract

The Severe acute respiratory syndrome coronavirus 2 (SARS-CoV-2) caused the “COVID-19” disease that has been declared by WHO as a global emergency. The pandemic, which emerged in China and widespread all over the world, has no specific treatment till now. The reported antiviral activities of isoflavonoids encouraged us to find out its in silico anti-SARS-CoV-2 activity. In this work, molecular docking studies were carried out to investigate the interaction of fifty-nine isoflavonoids against hACE2 and viral M^pro^. Several other in silico studies including physicochemical properties, ADMET and toxicity have been preceded. The results revealed that the examined isoflavonoids bound perfectly the hACE-2 with free binding energies ranging from −24.02 to −39.33 kcal mol^−1^, compared to the co-crystallized ligand (−21.39 kcal mol^–1^). Furthermore, such compounds bound the M^pro^ with unique binding modes showing free binding energies ranging from −32.19 to −50.79 kcal mol^–1^, comparing to the co-crystallized ligand (binding energy = −62.84 kcal mol^–1^). Compounds **33** and **56** showed the most acceptable affinities against hACE2. Compounds **30** and **53** showed the best docking results against M^pro^. In silico ADMET studies suggest that most compounds possess drug-likeness properties.

## 1. Introduction

In December 2019, an outbreak of severe pneumonia caused by the novel severe SARS-CoV-2 originated in Wuhan, China. The infection spread all over the world causing coronavirus disease (COVID-19) [1,2]. By October 2020, COVID-19 caused more than 33 million infections and more than 1 million deaths according to the WHO [3]. Unfortunately, till now there is no specific antiviral drug available for the treatment of COVID-19-infected people. However, some drugs such as remdesivir showed modest activity through decreasing the mortality rate and treatment time [4].

M^pro^ is an essential non-structural chymotrypsin-like cysteine proteases enzyme for the replication of coronavirus. It works on two large polyproteins (PP1a and PP1ab) releasing 16 essential non-structural proteins (NSPs 1-16) [5,6].

Angiotensin-converting enzyme (ACE-2) is a crucial enzyme in the renin-angiotensin system. It is a significant target for antihypertensive drugs [7]. It is primarily expressed in renal tubular epithelium and vascular endothelium cells [8]. It was also reported to be expressed in lungs and GIT, tissues shown to harbor SARS-CoV [9,10]. The binding of SARS-CoV-2 to ACE-2 receptors was reported to play a pivotal role in the first binding step at the cellular membrane [11]. SARS-CoV-2 entry was mediated by its transmembrane spike glycoprotein [12]. ACE-2 was identified as the cellular receptor to which spike glycoprotein of SARS-CoV-2 binds [13]. Several reports confirmed that the SARS-CoV-2 infects human cells through ACE-2 receptor [11,14]. Furthermore, it was found that the overexpression of ACE-2 in a living cell facilitates virus entry [15].

Natural secondary metabolites are a major source of anti-infective drugs. These metabolites could be originated from plants [16,17], marine [18,19], or microbial sources [20,21,22], and found to belong to various types such as saponins [23,24], alkaloids [25], pyrones [26], isochromenes [27], diterpenes[28], flavonoids [29,30], and isoflavonoids [31].

The isoflavonoids are an important polyphenolic subclass of the flavonoids with a skeleton based on a 3-phenylchroman structure [32]. The antiviral power of several isoflavonoid secondary metabolites has been proven in several scientific reports before. Torvanol A is a sulfated isoflavonoid isolated from the fruits of *Solanum torvum* and exhibited antiviral activity against herpes simplex virus type 1 with an IC_50_ value of 9.6 μg mL^−1^ [33]. Genistein; the major isoflavonoid of soybean seeds inhibited HSV-1 (KOS and 29R strains), and HSV-2 (333 strain) replications with IC_50_ values of 14.02, 7.76 and 14.12, respectively. In addition, three isoflavone glycosides were obtained from some hypocotyls of soybean seeds and could completely inhibit HIV-induced cytopathic effects and virus-specific antigen expression just six days after infection at a concentration of 0.25 mg mL^−1^ [34]. Daidzein was reported to inhibit the influenza virus at an IC_50_ of 51.2 µM [35].

**Figure 1:**
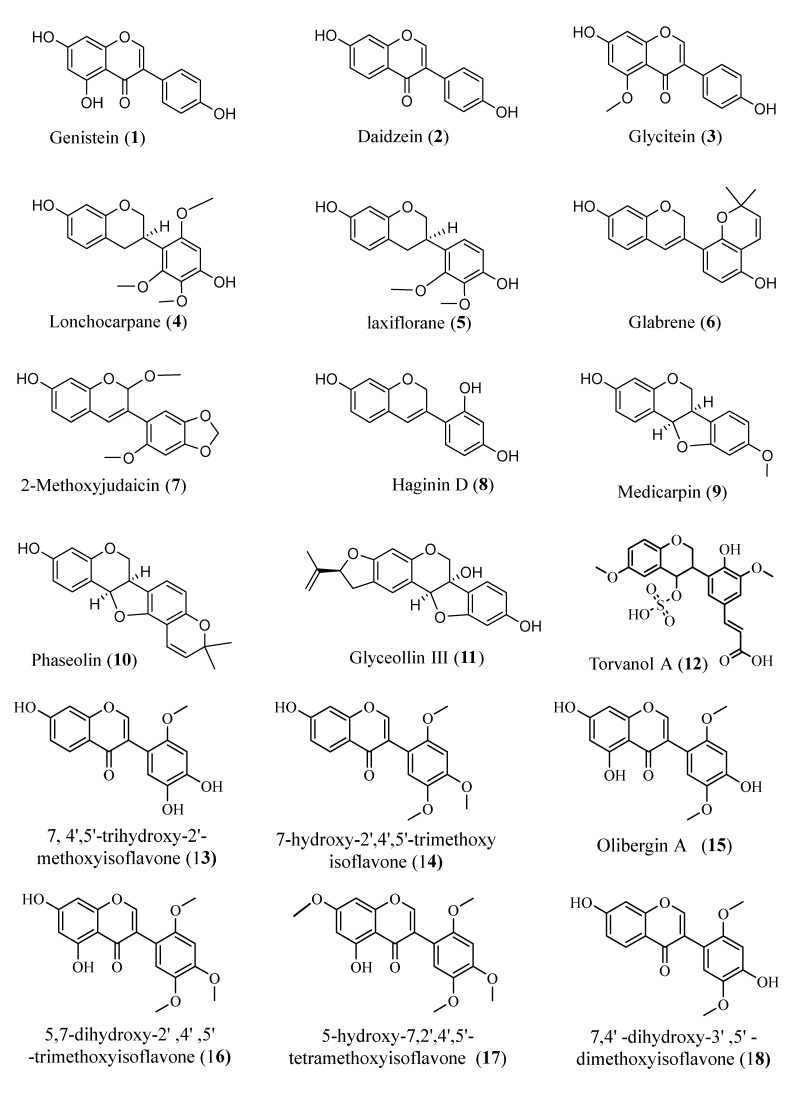

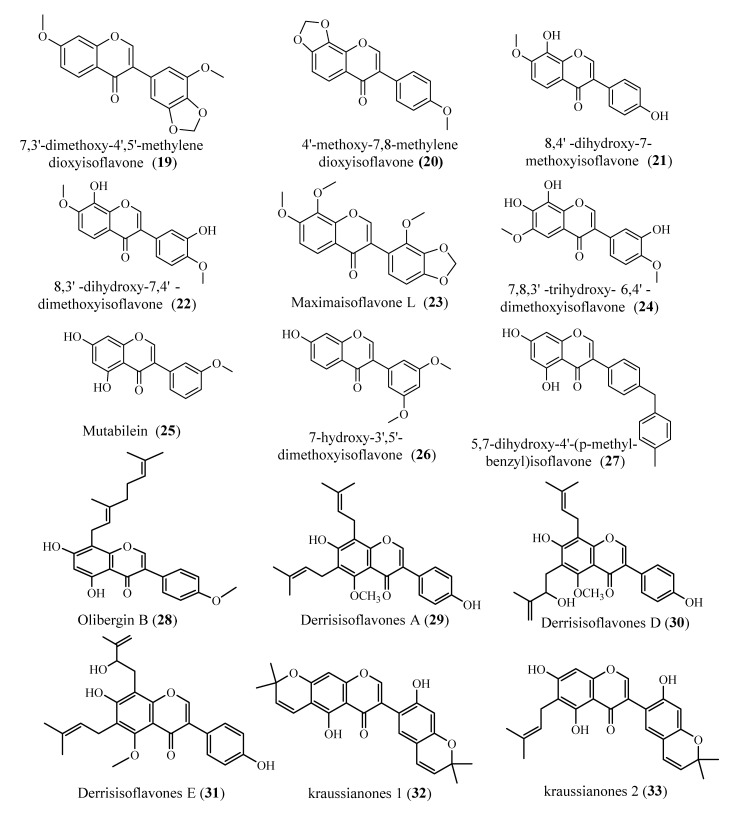

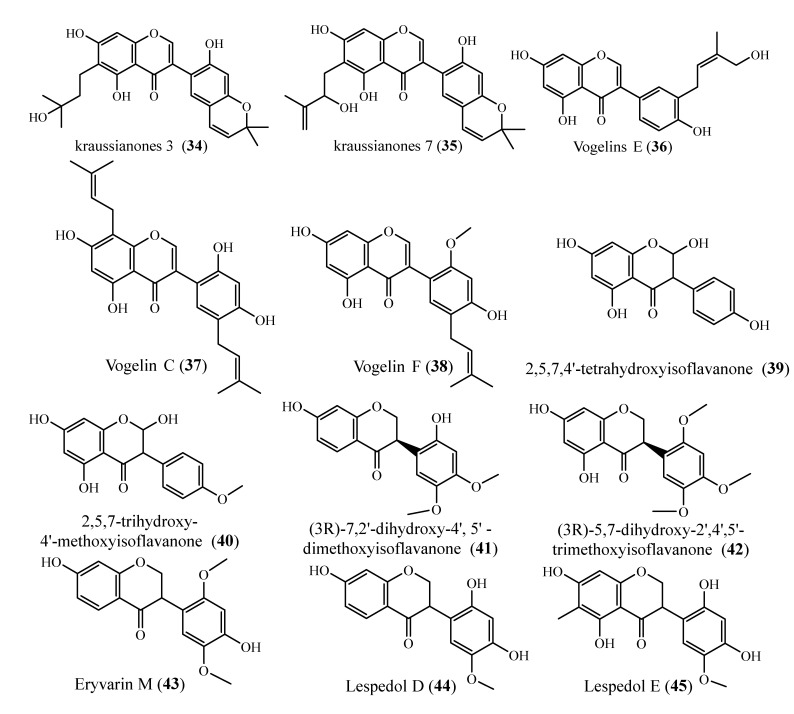

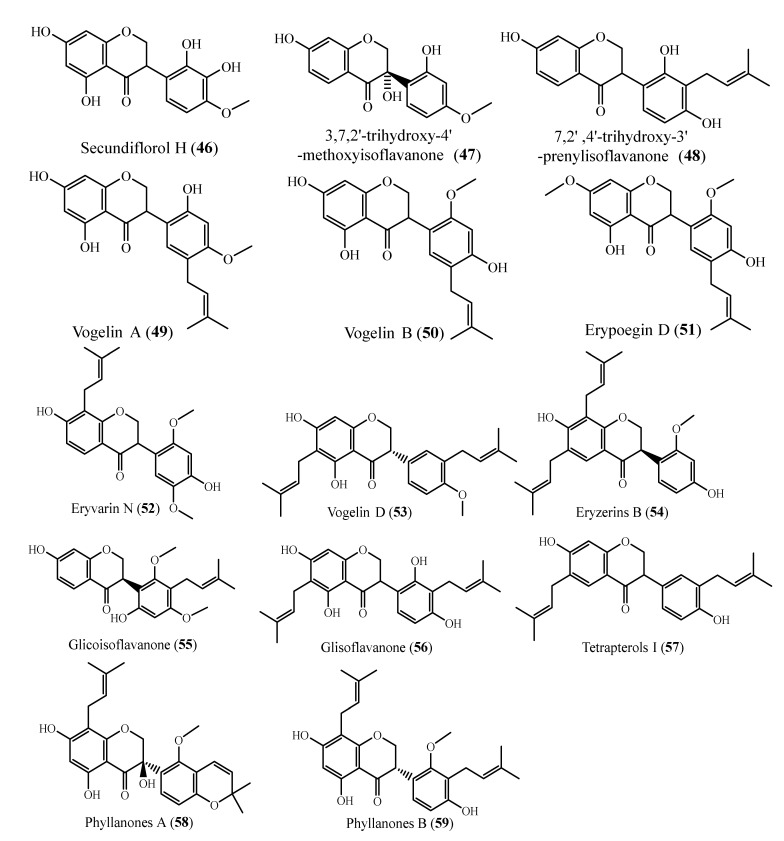
Structures of the examined isoflavonoids.

Furthermore, homoisoflavonoids showed great antiviral activity against the enteroviruses, Coxsackievirus B1, B3, B4, A9 and echovirus 30 [36]. Interestingly, a group of synthesized substituted homo-isoflavonoids exhibited promising inhibitory effects against human rhinovirus (HRV) 1B and 14 [37]. These findings inspired us to explore the potential of fifty-nine isoflavonoids (**1**–**59**) (Figure 1) as a possible treatment for COVID-19 through in silico examination of their potential to bind with ACE-2 and M^pro^ receptors.

## 2. Experimental

Drug-likeness properties were calculated using Lipinski’s rule of five, which suggested that the absorption of an orally administered compound is more likely to be better if the molecule satisfies at least three of the following rules: (i) H bond donors (OH, NH, and SH) ≤5; (ii) H bond acceptors (N, O, and S atoms) ≤10; (iii) molecular weight <500; (iv) logP <5. Compounds violating more than one of these rules could not have good oral bioavailability [38]. The pharmacokinetic properties (ADMET) of isoflavonoids and adherence with Lipinski’s rule of five were calculated using Discovery studio 4.0 software(Accelrys software Inc., San Diego, CA, USA) [39].

The title molecules were investigated with the aid of docking studies using *Discovery Studio 4.0* software (Accelrys software Inc., San Diego, CA, USA) for their binding capabilities against ACE-2 and M^pro^. The crystal structures of the target proteins were acquired from the RCSB Protein Data Bank (PDB ID: 6LZG, resolution: 2.50 Å [40] and 6LU7, resolution: 2.16 Å [41] for ACE-2 and M^pro^_,_ respectively). the co-crystallized ligands 2-acetamido-2-deoxy-*β*-D-glucopyranose (NAG) and *N*-[(5-methylisoxazol-3-yl)carbonyl]alanyl-l-valyl-*N*~1~-((1R,2Z)-4-(benzyloxy)-4-oxo-1-{[(3R)-2-oxopyrrolidin-3-yl]methyl}but-2-enyl)-l-leucinamide (N3) were used as reference molecules against hACE-2 and M^pro^_,_ respectively.

At first, water molecules were removed from the complex. Using the valence monitor method, the incorrect valence atoms were corrected. The energy minimization was then accomplished through the application of force fields CHARMM and MMFF94 [42,43,44,45]. The binding sites were defined and prepared for docking processes. Structures of the tested isoflavonoids and the co-crystallized ligands were sketched using ChemBioDraw Ultra 14.0 (PerkinElmer, Waltham, MA, USA) [46] and saved as MDL-SD files. Next, the MDL-SD files were opened, 3D structures were protonated, and energy was minimized by implementing force fields CHARMM and MMFF94, then adjusted for docking. CDOCKER protocol was used for docking studies using CHARMM-based molecular dynamics (MD) to dock the co-crystallized ligands into a receptor binding site [47,48]. In the docking studies, a total of 10 conformers were considered for each molecule. Finally, according to the minimum free energy of binding interaction, the most ideal pose was chosen.

The toxicity parameters for the examined compounds were calculated using *Discovery studio 4.0* software (Accelrys software Inc., San Diego, CA, USA). Simeprevir was used as a reference drug. At first, the CHARMM force field was applied then the compounds were prepared and minimized according to the preparation of small molecule protocol. Then different parameters were calculated from the toxicity prediction (extensible) protocol.

## 3. Results and Discussion

### 3.1. Pharmacokinetic Profiling Study

#### 3.1.1. Lipinski’s Rule of Five

In the present study, an in silico computational study of compounds (**1**–**59**) was performed to determine their physicochemical properties according to the directions of Lipinski’s rule of five [38] (Table 1).

It was found that almost all the tested isoflavonoids followed Lipinski’s rule of five and hence display a drug-like molecular (DLM) nature. The Log P, molecular weight, number of H-bond donors and number of H-bond acceptors of all isoflavonoids are within the accepted values (less than 5, 500, 5 and 10, respectively). with exceptions of compounds (**28**, **29**, **53**, **54** and **57**) that have log *p* values of 6.04, 6.07, 6.19, 6.19 and 6.21, respectively.

#### 3.1.2. ADMET Studies

*Discovery studio 4.0* software was used to predict ADMET descriptors (absorption, distribution, metabolism, excretion and toxicity) for the selected isoflavonoids using remdesivir as a reference drug. The predicted ADMET parameters of the tested compounds were listed in Table 2. The BBB penetration levels of **6**, **10** and **27** were expected to be high. On the other hand, the expected BBB penetration levels of all other isoflavonoids were ranging from medium to low. These results indicate that most of the tested compounds would be less likely to penetrate the CNS.

The plasma protein binding model predicts the binding ability of a ligand with plasma proteins which affects its efficiency. The results revealed that compounds **1**, **2**, **5**, **9**, **10**, **14**, **15**, **18**, **19**, **23**–**31**, **37**, **41**, **42**, **44**, **45**, **49**, **52**–**54**, **56**, **57** and **59** were expected to bind plasma protein by more than 95%, while **3, 8**, **13**, **16**, **17**, **20**–**22**, **33**, **39**, **40**, **48**, **50**, **51**, **55** and **58** showed a binding pattern of more than 90%. Contrastingly, compounds **4**, **6**, **7**, **11**, **12**, **32**, **34**, **35**, **38**, **43**, **46** and **47** were expected to bind the plasma protein less than 90%.

**Figure 2:**
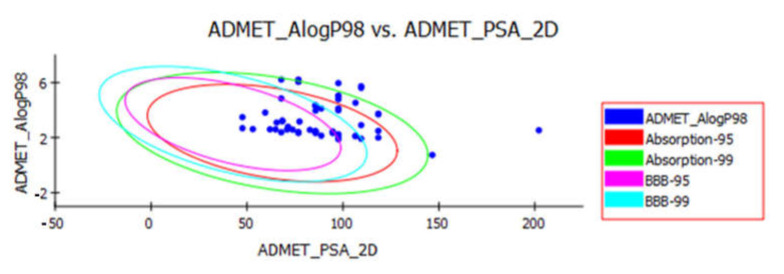
The expected ADMET study of the designed compounds and remdesivir.

Moreover, all the tested isoflavonoids were predicted to have good absorption behavior better than that of remdesivir. Also, the solubility levels of most compounds were expected to be in the good range (Table 2 and Figure 2).

### 3.2. Molecular Docking

#### 3.2.1. Validation Process

**Figure 3:**
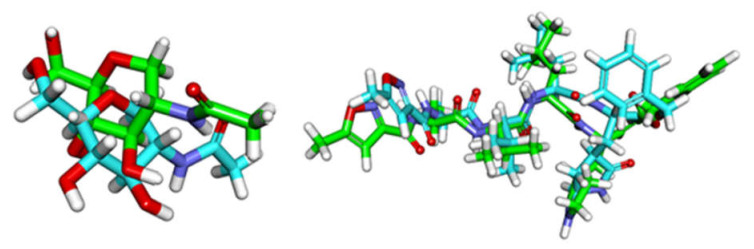
Superimposition of the co-crystallized poses (green) and the docking pose (maroon) of the same ligands. (**Left**): hACE2 (RMSD = 2.4 Å), (**right**): M^pro^ (RMSD = 2.8 Å).

Validation of the docking procedures was achieved via re-docking of the co-crystallized ligands against the active pocket of hACE2 and M^pro^. The calculated RMSD values between the re-docked poses and the co-crystallized ones were 2.4 and 2.8 Å. Such values of RMSD indicated the efficiency and validity of the docking processes (Figure 3).

#### 3.2.2. HACE2

Coronavirus spike receptor-binding domain complexed with its receptor hACE-2 (PDB: 6LZG) used as a target for the docking studies of selected isoflavonoids. The results demonstrated that all isoflavonoids bound strongly to hACE-2 with binding energies bitter than that of the co-crystallized ligand (NAG). This indicated that the affinity of the tested isoflavonoids toward hACE-2 is higher than that of the co-crystallized ligand (Table 3). Moreover, almost all the tested isoflavonoids exhibited binding modes similar to that of NAG.

**Figure 4:**
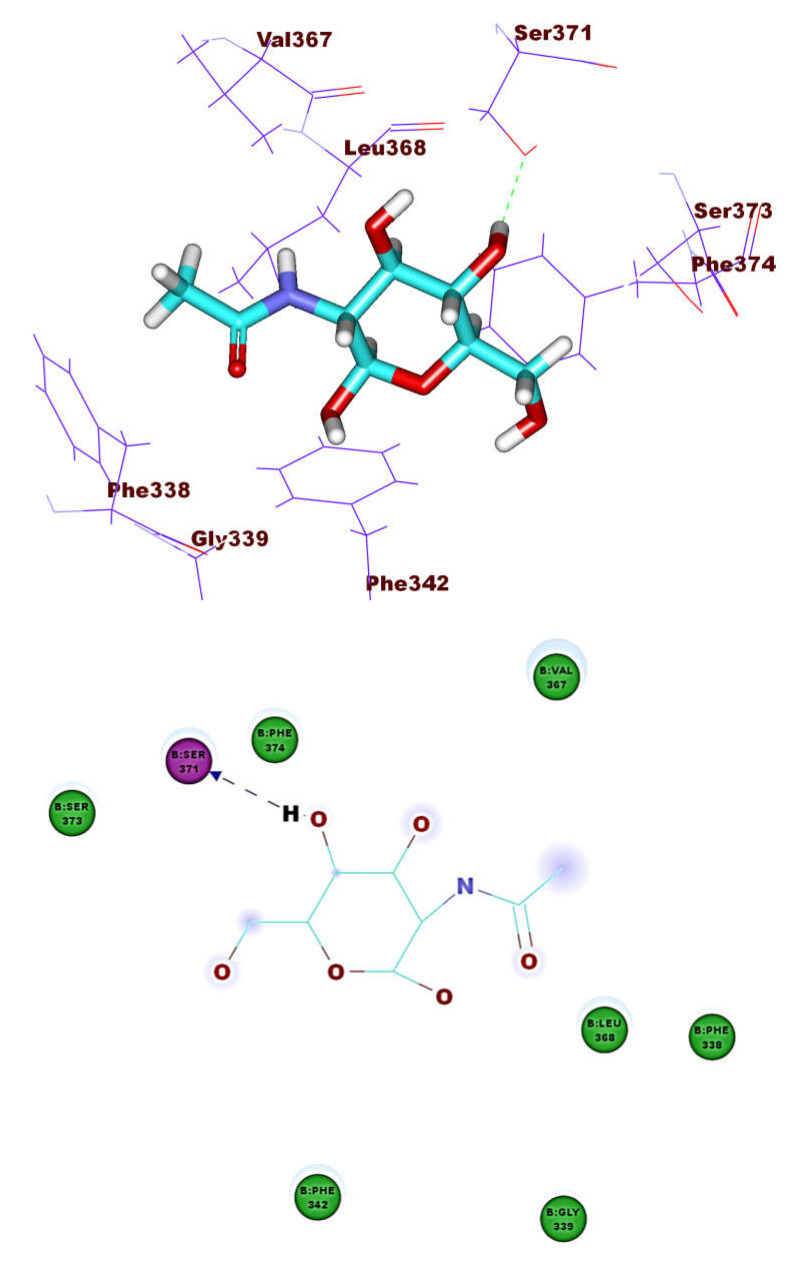
The co-crystallized ligand (NAG) docked into ACE-2, forming one H. bond with Ser371.

**Figure 5:**
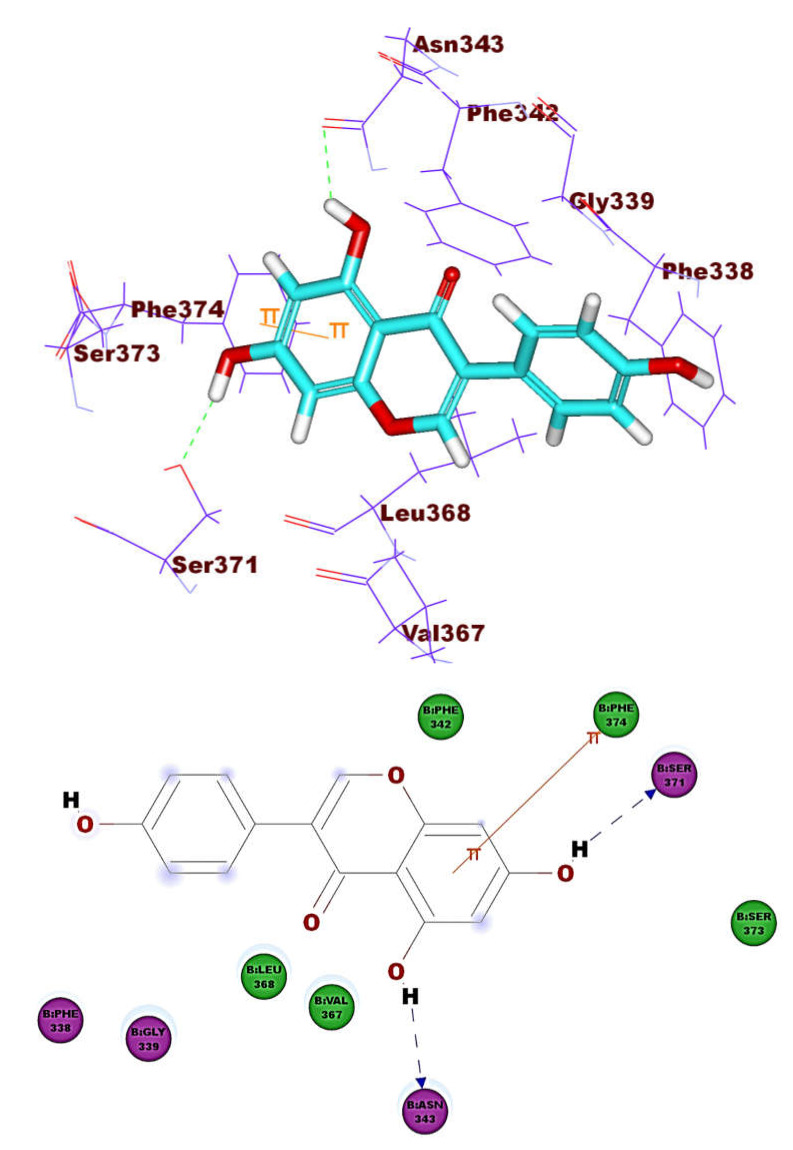
Compound **1** docked into ACE-2, forming two H. bonds with Ser371 and Asn343.

**Figure 6:**
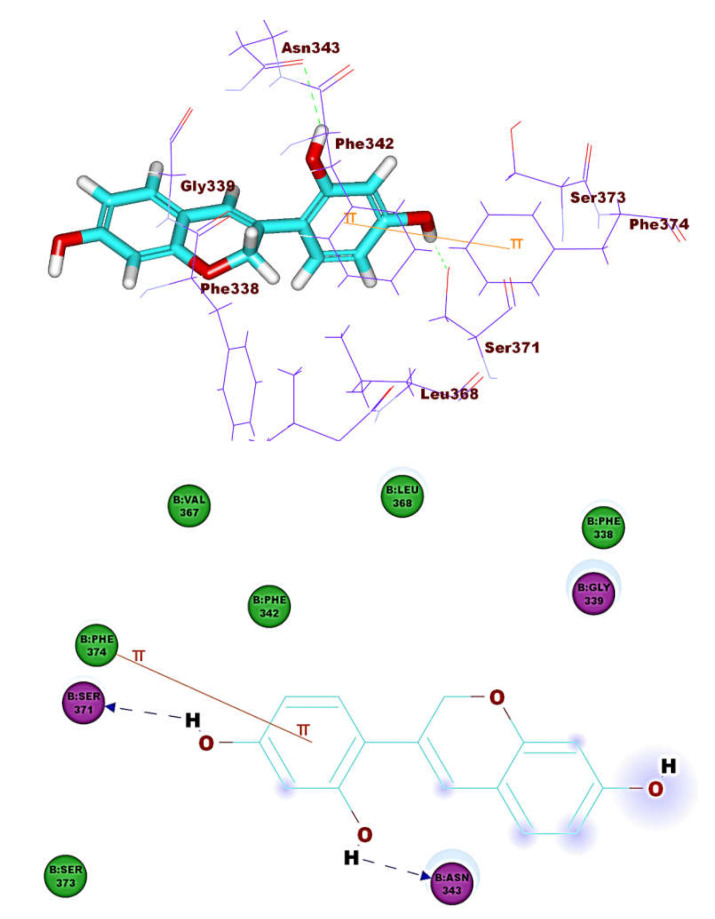
Comound **8** docked into ACE-2, forming two H. bonds with Ser371 and Asn343.

**Figure 7:**
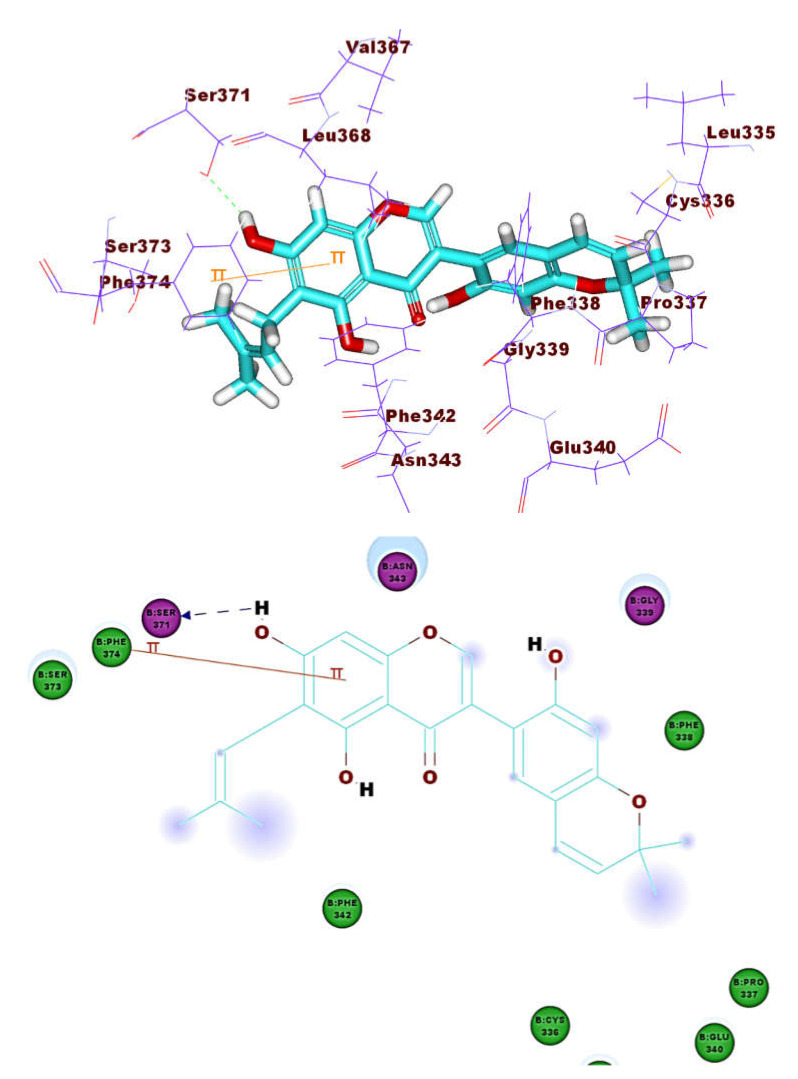
Compound **33** docked into ACE-2, forming one H. bond with Ser371.

**Figure 8:**
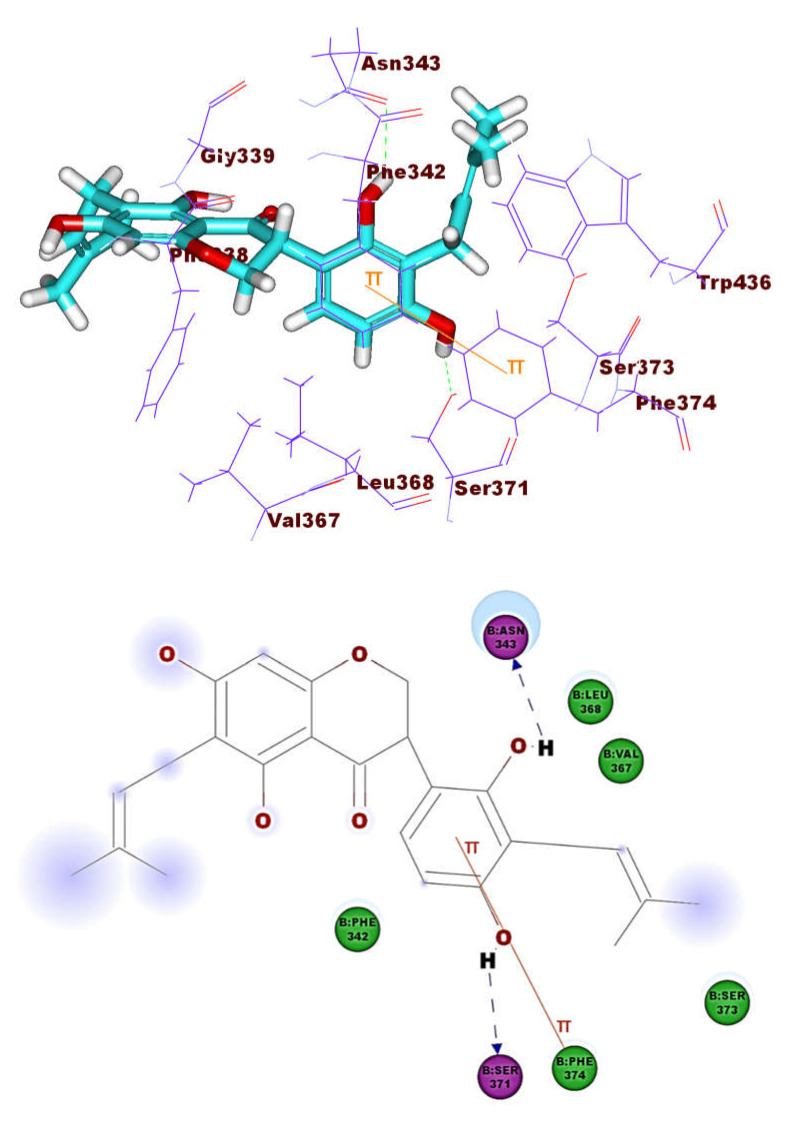
Compound **56** docked into ACE-2, forming two H. bonds with Ser371 and Cys336.

The binding pattern of co-crystallized ligand (NAG) demonstrated single hydrogen bonding interaction with Ser371 residue (Figure 4). NAG showed binding energies of −21.39 kcal mol^−1^. It was found that most of the tested isoflavonoids exhibited binding modes similar to the reference molecule. Compounds **1** (−30.90 kcal mol^–1^) and **8** (−27.41 kcal mol^−1)^ demonstrated an additional hydrogen bond with Asn343 residue (Figure 5 and Figure 6). This extra hydrogen bond may account for the relatively high binding affinity of both compounds. Furthermore, compounds **33** (Figure 7) and **56** (Figure 8) were found to have good binding energy values of −36.35 and −34.90 kcal mol^−1^, respectively. Compound **33** formed a binding mode like that of the reference ligand as it formed one hydrogen bond with Ser371 and seven hydrophobic interactions with Phe374, Phe342, Ser371, Asn343, Cys336, Glu340, and Ser373. Interestingly, compound **56** formed two hydrogen bonds with Ser371 and Cys336 in addition to seven hydrophobic interactions with Phe374, Phe338, Ser371, Val367, Cys336, Leu368, and Ser373.

Such results indicate the significance of the tested isoflavonoids as potential inhibitors for hACE-2. Consequently, such compounds may inhibit the entrance of coronavirus into human cells.

#### 3.2.3. Main Protease (M^pro^)

The docking results of isoflavonoids into the active site of coronavirus M^pro^ (PDB: 6LU7) were listed in Table 4. The results showed that all tested isoflavonoids can bind to M^pro^ with one or more hydrogen bonds. At the same time, the tested compounds bound to the receptor with free binding energies ranging from −32.19 to −50.79 kcal mol^−1^, compared to the co-crystallized (binding energy = −62.84 kcal mol^−1^).

**Figure 9:**
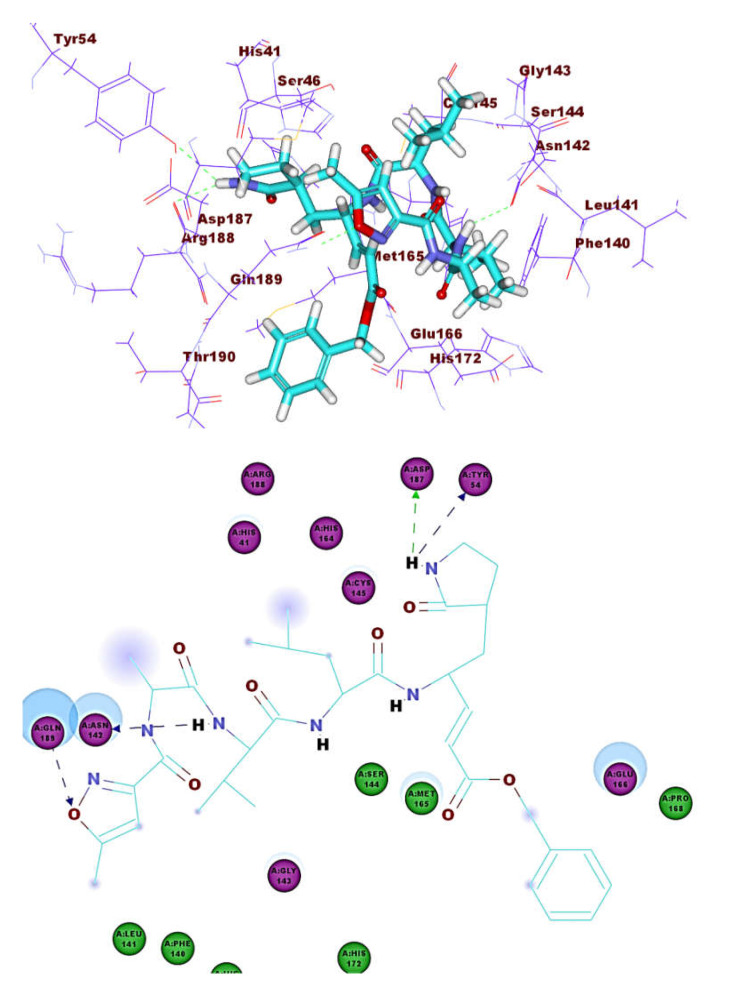
The co-crystallized ligand (N3) docked into M^pro^, forming four H. bonds with Gln189, Tyr 54, Asp 142, Asp187.

**Figure 10:**
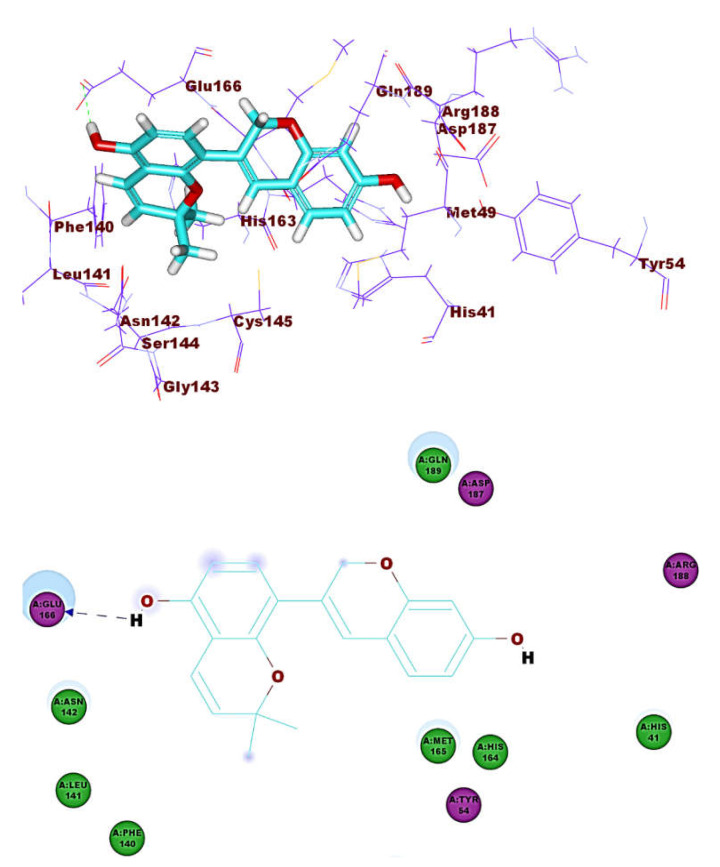
Compound **6** docked into M^pro^, forming one H. bond with Glu166.

**Figure 11:**
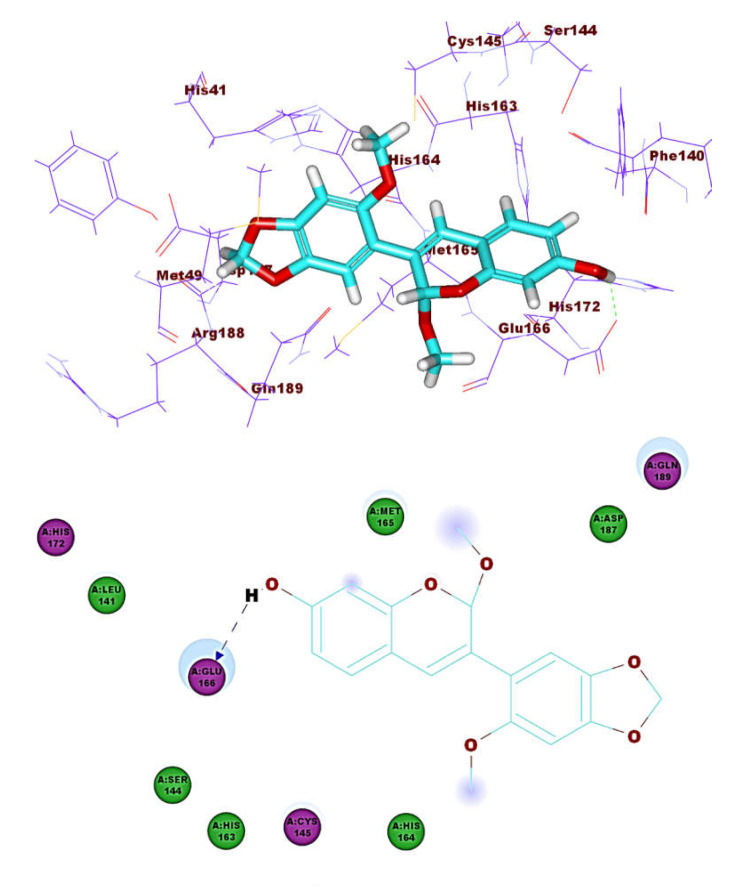
Compound **7** docked into M^pro^, forming one H. bond with Glu166.

**Figure 12:**
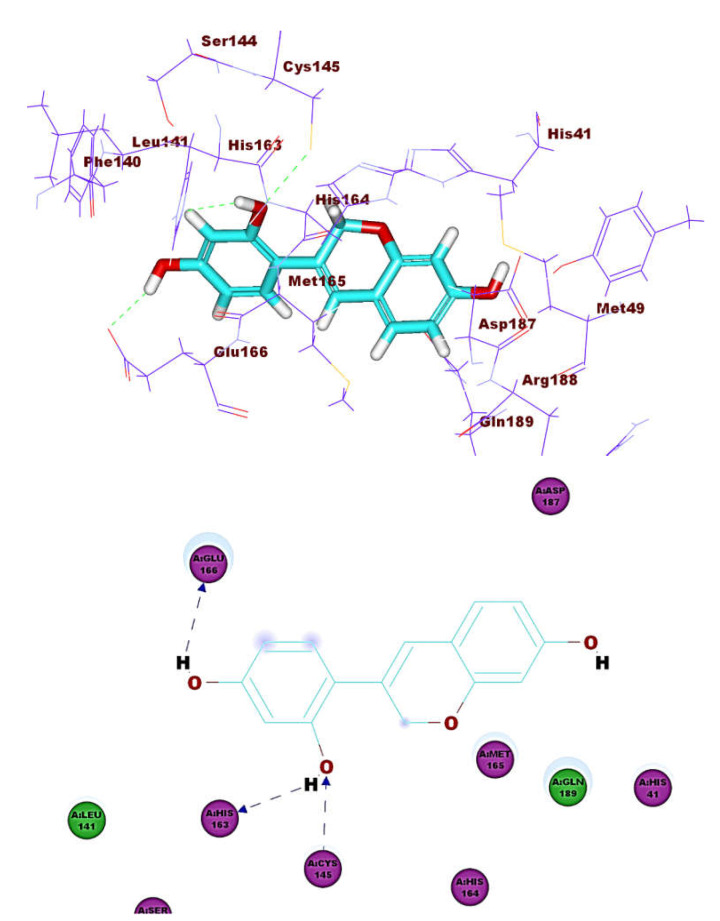
Compound **8** docked into M^pro^, forming three H. bonds with Glu166, Cys145 and His163.

**Figure 13:**
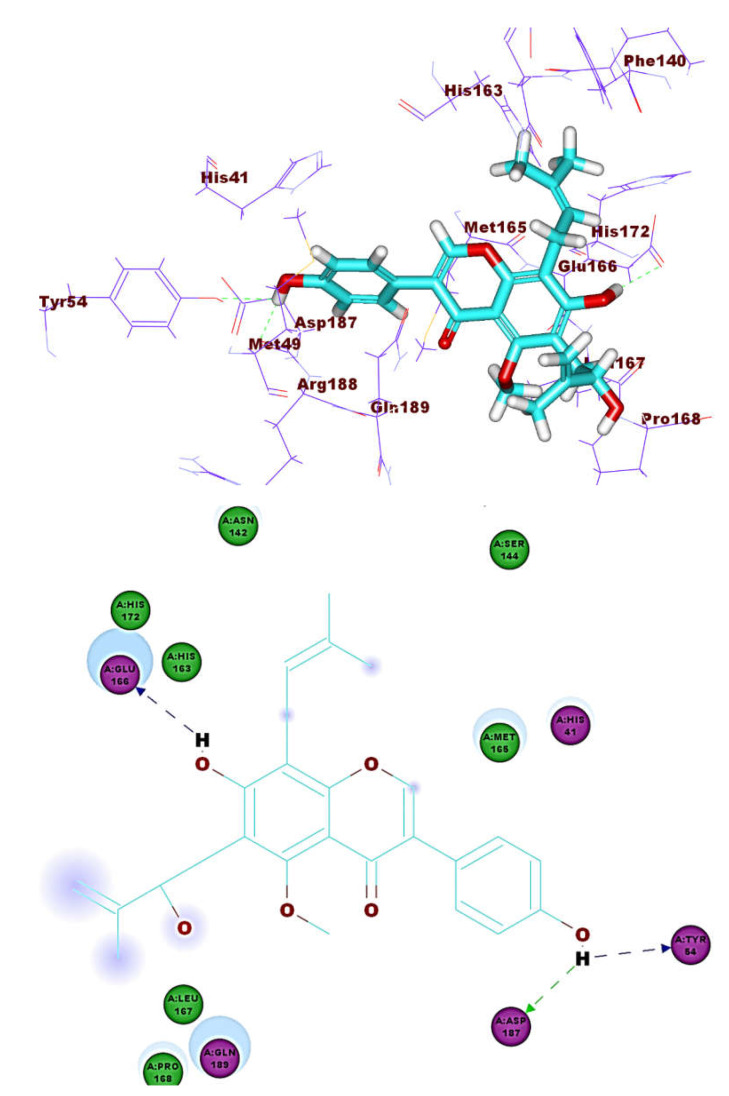
Compound **30** docked into M^pro^, forming three H. bonds with Glu166, Cys145 and His163.

**Figure 14:**
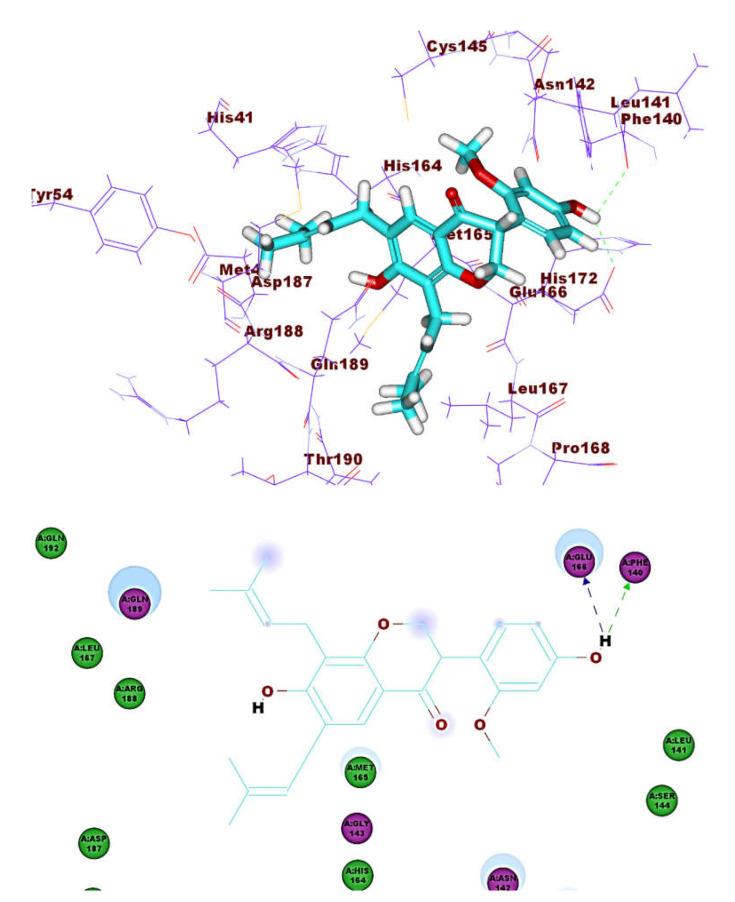
Compound **53** docked into M^pro^, forming two H. bonds with Glu166, Phe140.

These results revealed that the affinities of the presented isoflavonoids against M^pro^ are lower than that of N3. Despite that, the binding energies are still considerable, and their binding modes are great which making these isoflavonoids seem to be biologically active ligands to some extent. Figure 9, Figure 10, Figure 11, Figure 12, Figure 13 and Figure 14 illustrate the binding patterns of N3, compound **6** (binding energy = −41.41 kcal mol^−1^), compound **7** (binding energy = −40.11 kcal mol^−1^), compound **8** (binding energy = −42.73 kcal mol^−1^), compound **30** (binding energy = −48.39 kcal mol^−1^), and compound **53** (binding energy = −46.90 kcal mol^−1^), respectively.

Compound **30** formed a binding mode like that of the reference ligand as it formed three hydrogen bonds with Glu166, Tyr54, and Asp187. Furthermore, it formed eight hydrophobic interactions with His41, Gln189, His163, Met165, Tyr54, Asp187, Leu167, and Glu166. For compound **53,** it formed two hydrogen bonds with Glu166, Phe140. Besides, it formed six hydrophobic interactions with Glu166, Gln189, Leu141, Met165, His172, and Phe140.

#### 3.2.4. Structure-Activity Relationship (SAR)

**Figure 15:**
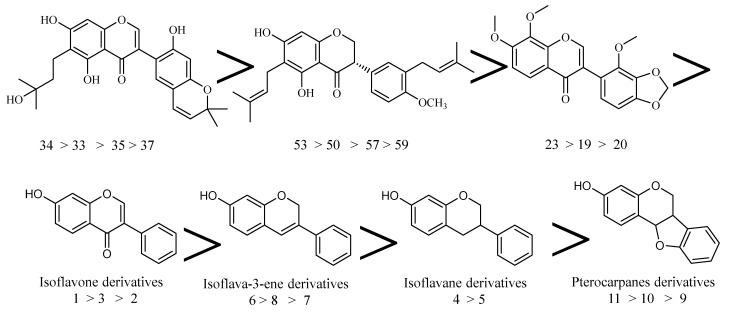
Schematic diagram showing the different affinities of isoflavonoids against hACE-2.

Based on the binding affinities of the tested compounds against hACE-2, we can obtain valuable SAR. Generally, the tested compounds showed decreased affinity against hACE-2 in descending order of isoflavone derivatives (compounds **34**, **33**, **35** and **37**) > isoflavane derivatives (compounds **50**, **53**, **57** and **59**) > isoflavone derivatives (compounds **19**, **20** and **23**) > isoflavone derivatives (compounds **1**–**3**) > isoflava-3-ene derivatives (compounds **6**–**8**) > isoflavane derivatives (compounds **4** & **5**) > pterocarpanes derivatives (compounds **9**–**11**) (Figure 15).

For isoflavone derivatives (compounds **34**, **33**, **35** and **37**), it was found that compound **34** incorporating 3-hydroxy-3-methylbutyl moiety at 6-position was more active that compound **33** incorporating 3-methylbut-2-en-1-yl moiety at the same position. The latter was more active than compound **35** incorporating 2-hydroxy-3-methylbut-3-en-1-yl moiety at the same position. Compound **37** incorporating 3-methylbut-2-en-1-yl moiety at 8-position was less active than the corresponding members.

With regard to isoflavane derivatives (compounds **50**, **53**, 57 and **59**), it was found that 3-methylbut-2-en-1-yl moiety is critical for binding affinity. compound **50** incorporating this moiety at 3- and 6-positions of 4-chromanone nucleus was more active than compound **53** incorporating this moiety at 5-position of phenyl ring. The latter was more active than compound **57** incorporating 3-methylbut-2-en-1-yl moiety at both 6-position position of 4-chromanone nucleus and 3-position of phenyl ring. Compound **59** incorporating 3-methylbut-2-en-1-yl moiety at both 8-position position of 4-chromanone nucleus and 3-position of phenyl ring was less active than the corresponding members.

Regarding isoflavone derivatives (compounds **19, 20,** and **23**), it was found that the presence of 1,3-dioxole moiety can affect the affinity depending on it position. compound **19** incorporating 1,3-dioxole moiety at 3,4-positions of phenyl ring was more active than compound **20** incorporating this moiety at 7,8-position of 4H-chromen-4-one nucleus. The latter was more active than compound **23** incorporating 1,3-dioxole moiety at 4,5-position position of phenyl ring.

Then, we investigated the effect of substitutions at isoflavone derivatives on the binding affinity. It was found that the substitutions at 5-position with hydroxyl (compound **1**) and methoxy (compound **3**) group, increase the binding of isoflavones against hACE-2, with an increased affinity of hydroxyl derivative.

Regarding the effect of substitutions at isoflava-3-ene, it was found that the derivative with additional pyran ring (compound **6**) was more active than the corresponding member with free OH group at position-1 of phenyl ring (compound **8**) which was more potent than compound **7** incorporating a dioxolan ring**_._**

Observing binding affinities of isoflavane derivatives. It was found that compound **4** incorporating an additional methoxy group at 6 position of phenyl ring showed better binding affinity against hACE-2 than the unsubstituted derivative (compound **5**). Such a result may be attributed to the electron donating effect of the methoxy group.

Concerning the activity of different pterocarpan derivatives, it was noted that compound **11**, which contained an additional tetrahydrofuran ring attached to the chromene ring, showed better binding affinity inside the hACE-2 than compounds **9** and **10**, which contained free OH groups at the chromene ring.

### 3.3. Toxicity Studies

Toxicity prediction was carried out based on the validated and constructed models in *Discovery studio 4.0* software [49,50] as follows. (i) FDA rodent carcinogenicity test which computes the probability of a compound to be a carcinogen. (ii) Carcinogenic potency TD_50_ which predicts the tumorigenic dose rate 50 (TD_50_) of a drug in a rodent chronic exposure toxicity test of carcinogenic potency [51]. (iii) Rat maximum tolerated dose (MTD) [52,53]. (iv) Rat oral LD_50_ which predicts the rat oral acute median lethal dose (LD_50_) of a chemical [54]. (v) Rat chronic LOAEL which predicts the rat chronic lowest observed adverse effect level (LOAEL) value [55,56]. (vi) Ocular irritancy predicts whether a particular compound is likely to be an ocular irritant and how severe the irritation is in the Draize test [57]. (vii) Skin irritancy predicts whether a particular compound is likely to be a skin irritant and how severe it is in a rabbit skin irritancy test [57].

As shown in Table 5, most compounds showed in silico low toxicity against the tested models. FDA rodent carcinogenicity model indicated that most of the tested compounds are non-carcinogens. Only compounds **6**, **9**, and **10** were predicted to be carcinogens so that, these compounds do not have the likeness to be used as drugs.

For the carcinogenic potency TD_50_ rat model, the examined compounds showed TD_50_ values ranging from 0.44 to 322.42 mg Kg^−1^ body weight/day which are higher than simeprevir (0.280 mg Kg^−1^ body weight/day).

Regarding the rat MTD model, the compounds showed MTD with a range of 0.061 to 0.764 g kg^−1^ body weight higher than simeprevir **(**0.003 g kg^−1^ body weight).

Concerning the rat oral LD_50_ model, the tested compounds showed oral LD_50_ values ranging from 0.10 to 4.66 mg Kg^−1^ body weight/day), while simeprevir exhibited an oral LD_50_ value of 0.21 mg Kg^–1^ body weight/day. About the rat chronic LOAEL model, the compounds showed LOAEL values ranging from 0.004 to 0.865 g kg^−1^ body weight. These values are higher than simeprevir (0.002 g kg^−1^ body weight). Moreover, most of the compounds were predicted to be irritant against the ocular irritancy model. On the other hand, the tested compounds were predicted to be mild or non-irritant against the skin irritancy model.

## 4. Conclusions

There is an urgent global need to find a cure for COVID-19. The present work is an attempt to find some natural compounds with potential activity against COVID-19. Accordingly, docking studies were carried out for fifty-nine isoflavonoid derivatives against two essential targets (hACE-2 and M^pro^). The obtained results showed that the tested isoflavonoids can strongly bind the hACE-2 and M^pro^ with great binding modes. Based on in silico studies, SARs were established. SAR studies afforded an insight into the pharmacophoric groups which may serve as a guide for the design of new potential anti-COVID-19 agents. Generally, the tested compounds showed decreased affinity against hACE-2 in descending order of isoflavone derivatives (compounds **33**–**35** and **37**) > isoflavane derivatives (compounds **50**, **53**, **57** and **59**) > isoflavone derivatives (compounds **19**, **20** and **23**) > isoflavone derivatives (compounds **1**–**3**) > isoflava-3-ene derivatives (compounds **6**–**8**) > isoflavane derivatives (compounds **4** and **5**) > pterocarpan derivatives (compounds **9**–**11**) Finally, compounds **33** and **56** showed the most acceptable affinity against hACE2; compounds **30** and **53** showed the best docking results against M^pro^. In addition, these compounds showed good physicochemical and cytotoxicity profiles. Moreover, in silico investigation of physicochemical properties, ADMET and toxicity studies revealed good properties and general low toxicity. Consequently, this study strongly suggests in vitro and in vivo studies for the most active isoflavonoids against COVID-19**.**

## Figures and Tables

**Table 1:** physicochemical properties of the tested isoflavonoids.

Comp.	Lipinski’s Rule of Five			
	Log P ^a^	Molecular Wight	HBD ^b^	HBA ^c^
**1**	2.14	270.23	3	5
**2**	2.38	254.23	2	4
**3**	2.36	284.26	2	5
**4**	3.16	332.34	2	6
**5**	3.17	302.32	2	5
**6**	3.81	322.35	2	4
**7**	3.09	328.31	1	6
**8**	2.78	256.25	3	4
**9**	2.68	270.28	1	4
**10**	3.48	322.35	1	4
**11**	3.23	338.35	2	5
**12**	−1.50	450.41	1	10
**13**	2.12	300.26	3	6
**14**	2.57	328.31	1	6
**15**	2.10	330.28	3	7
**16**	2.33	344.31	2	7
**17**	2.55	358.34	1	7
**18**	2.34	314.28	2	6
**19**	2.60	326.3	0	6
**20**	2.61	296.27	0	5
**21**	2.36	284.26	2	5
**22**	2.34	314.28	2	6
**23**	2.58	356.32	0	7
**24**	2.10	330.28	3	7
**25**	2.36	284.26	2	5
**26**	2.59	298.2	1	5
**27**	4.84	358.38	2	4
**28**	6.04	420.49	2	5
**29**	6.07	420.49	2	5
**30**	5.03	436.49	3	6
**31**	5.03	436.49	3	6
**32**	3.95	418.43	2	6
**33**	4.78	420.45	3	6
**34**	3.68	438.4	4	7
**35**	3.73	436.45	4	7
**36**	2.90	354.35	4	6
**37**	5.61	422.4	4	6
**38**	3.9	368.38	3	6
**39**	1.91	288.25	4	6
**40**	2.14	302.27	3	6
**41**	2.46	316.3	2	6
**42**	2.44	346.33	2	7
**43**	2.465	316.3	2	6
**44**	2.24	302.27	3	6
**45**	2.48	332.3	4	7
**46**	1.99	318.278	4	7
**47**	1.88	302.27	3	6
**48**	4.11	340.37	3	5
**49**	4.09	370.39	3	6
**50**	4.09	370.39	3	6
**51**	4.32	384.422	2	6
**52**	4.32	384.42	2	6
**53**	6.19	422.51	2	5
**54**	6.19	422.51	2	5
**55**	4.32	384.42	2	6
**56**	5.72	424.48	4	6
**57**	6.21	392.48	2	4
**58**	4.52	452.49	3	7
**59**	5.95	438.51	3	6

^a^ Partition coefficient; ^b^ Hydrogen bond donors; ^c^ Hydrogen bond acceptors.

**Table 2:** Predicted ADMET descriptors for the tested isoflavonoids and remdesivir.

Compound	BBB Level ^a^	Absorption Level ^b^	PPB ^c^	Solubility Level ^d^
**1**	3	0	2	3
**2**	2	0	2	3
**3**	3	0	1	3
**4**	2	0	0	3
**5**	2	0	2	3
**6**	1	0	0	2
**7**	2	0	0	2
**8**	2	0	1	3
**9**	2	0	2	3
**10**	1	0	2	2
**11**	2	0	0	2
**12**	4	2	0	4
**13**	3	0	1	3
**14**	3	0	2	3
**15**	3	0	2	3
**16**	3	0	1	3
**17**	3	0	1	3
**18**	3	0	2	3
**19**	2	0	2	2
**20**	2	0	1	2
**21**	3	0	1	3
**22**	3	0	1	3
**23**	2	0	2	2
**24**	3	0	2	3
**25**	3	0	2	3
**26**	2	0	2	3
**27**	1	0	2	2
**28**	4	1	2	2
**29**	4	1	2	1
**30**	4	1	2	2
**31**	4	1	2	2
**32**	2	0	0	2
**33**	4	1	1	2
**34**	4	1	0	2
**35**	4	1	0	2
**36**	4	0	1	3
**37**	4	2	2	2
**38**	4	0	0	2
**39**	3	0	1	3
**40**	3	0	1	3
**41**	3	0	2	3
**42**	3	0	2	3
**43**	3	0	0	3
**44**	3	0	2	3
**45**	4	0	2	3
**46**	4	0	0	3
**47**	3	0	0	3
**48**	2	0	1	2
**49**	4	0	2	2
**50**	4	0	1	2
**51**	2	0	1	2
**52**	2	0	2	2
**53**	4	2	2	1
**54**	4	2	2	1
**55**	2	0	1	2
**56**	4	2	2	2
**57**	4	1	2	1
**58**	4	1	1	2
**59**		2	2	2
**Remdesivir**	4	3	0	2

^a^ BBB level, blood brain barrier level, 0 = very high, 1 = high, 2 = medium, 3 = low, 4 = very low. ^b^ Absorption level, 0 = good, 1 = moderate, 2 = poor, 3 = very poor. ^c^ PBB, plasma protein binding, 0 means less than 90%, 1 means more than 90%, 2 means more than 95%. ^d^ solubility level, 0 = extremely low, 1 = very low, 2 = low, 3 = good, 4 = optimal.

**Table 3:** Free binding energies of the selected isoflavonoids and the co-crystallized ligand (NAG) against hACE-2 and amino acid residues involved in H. bonds and hydrophobic interaction.

Comp.	Binding Energy (kcal mol^−1^)	No. of H. Bonds	Involved Amino Acid Residues	Amino Acid Residues Involved in Hydrophobic inTeraction
**1**	−30.90	2	Ser371, Asn343	Phe374, Gly339, Ser371, Phe338, Phe342
**2**	−27.84	1	Ser371	Phe338, Phe342, Gly339
**3**	−28.13	1	Ser371	Phe374, Phe342, Phe338, Gly339
**4**	−25.52	1	Ser371	Phe374, Phe342, Phe338, Gly339
**5**	−24.12	1	Ser371	Phe374, Phe342, Phe338, Leu368, Gly339
**6**	−26.14	1	Ser371	Phe342, Phe338, Phe374
**7**	−25.95	1	Ser371	Phe342, Phe338, Phe374
**8**	−27.41	2	Ser371, Asn343	Phe374, Phe342, Phe338
**9**	−22.32	1	Ser371	Phe374, Phe342, Phe338
**10**	−23.66	0	0	Phe374, Phe342, Phe338, Ser371, Gly339
**11**	−24.02	1	Ser371	Phe374, Phe342, Phe338
**12**	−31.01	2	Asp364	Phe338, Ser371, Leu368, Cys336, Phe374, Val367
**13**	−27.85	0	0	Asn343, Ser371, Leu368, Cys336, Phe374, Val367
**14**	−25.17	1	Cys336	Phe338, Ser371, Ser373, Leu368, Cys336, Phe374, Val367
**15**	−27.52	1	Cys336	Phe374, Phe342, Ser371, Leu368, Cys336, Val367
**16**	−27.42	1	Cys336	Ser371, Leu368, Cys336, Phe374, Val367, Gly339
**17**	−25.02	1	Trp436	Phe374, Leu368, Val367, Phe342
**18**	−23.37	1	Cys336	Phe338, Leu368, Cys336, Phe342, Val367, Asn343
**19**	−30.52	1	Gly339	Phe374, Phe338, Ser371, Gly339, Cys336, Leu368, Val367
**20**	−29.50	0	0	Phe374, Phe338, Ser371, Cys336, Leu368, Val367
**21**	−24.10	0	0	Phe338, Ser371, Cys336, Leu368, Val367, Phe374
**22**	−28.66	1	Cys336	Asn434, Phe338, Ser371, Cys336, Leu368, Val367
**23**	−33.20	0	0	Phe338, Ser371, Cys336, Leu368, Val367, Ser373
**24**	−32.74	2	Ser371, Cys336	Phe374, Phe338, Gly339, Cys336, Ser371, Leu368, Phe432
**25**	−24.43	1	Ser373	Gly339, Leu368, Phe338, Ser371, Cys336
**26**	−27.27	1	Ser373	Asn343, Gly339, Leu368, Phe338, Ser371, Cys336
**27**	−30.81	1	Cys336	Phe374, Phe338, Ser371, Cys336, Leu368, Val367, Phe342, Asn343
**28**	−29.91	0	0	Leu368, Val367, Phe342, Asn343, Cys336, Phe338, Ser371,
**29**	−32.76	2	Ser371, Cys336	Phe374, Phe338, Ser371, Val367, Cys336, Leu368, Ser373
**30**	−29.12	2	Asn343, Cys336	Phe338, Ser371, Gly339, Cys336, Leu368, Ser373, Asn343
**31**	−30.84	1	Asn364	Cys336, Leu368, Ser373, Asn343, Val362, Asn364
**32**	−33.95	0	0	Phe338, Val367, Cys336, Leu368, Ser373, Asn440, Asn364
**33**	−36.35	1	Ser371	Phe374, Phe342, Ser371, Asn343, Cys336, Glu340, Ser373
**34**	−39.33	1	Asp364	Phe338, Phe342, Asn343, Val367, Asp364, Cys336, Leu335, Leu386
**35**	−34.48	3	Cys336, Gly339, Glu340	Phe374, Phe338, Val367, Cys336, Leu368, Ser373, Gly339, Glu340
**36**	−34.80	2	Cys336, Gly339	Phe338, Leu335, Asn343, Ser373
**37**	−34.37	2	Ser371, Ser373	Leu368, Ser371, Asn343, Ser373, Phe338, Phe342
**38**	−30.09	2	Cys336, Gly339	Phe338, Leu335, Cys336, Gly339, Asn343, Ser373
**39**	−25.26	1	Ser371	Phe374, Phe338, Ser371, Val367, Cys336, Leu368, Ser373
**40**	−23.32	1	Ser373	Phe338, Val367, Cys336, Leu368, Ser373
**41**	−29.16	1	Cys336	Cys336, Phe338, Val367, Leu368, Ser373
**42**	−32.12	1	Ser371	Phe374, Val367, Cys336, Leu368, Ser373, Phe338, Ser371,
**43**	−27.79	1	Ser371	Phe374, Phe338, Ser371, Val367, Cys336, Leu368
**44**	−27.53	1	Ser373	Phe338, Phe374, Val367, Cys336, Leu368, Ser373
**45**	−31.39	1	Cys336	Cys336, Phe342, Val367, Leu368, Gly339, Asp364
**46**	−30.09	2	Cys336, Gly339	Phe338, Leu335, Cys336, Gly339, Asn343, Ser373
**47**	−25.11	0	0	Phe338, Asn343, Cys336, Leu368, Ser373
**48**	−34.79	1	Cys336	Cys336, Asn343, Phe338, Val367, Leu368, Ser373
**49**	−31.79	1	Cys336	Phe338, Asn343, Cys336, Leu368, Val367
**50**	–30.39	1	Cys336	Cys336, Phe338, Val367, Leu368, Ser373
**51**	−30.81	1	Ser371	Phe342, Asn343, Phe374, Ser371, Leu368
**52**	−29.33	1	Gly339	Phe338, Leu335, Cys336, Gly339, Val367, Asn343, Ser373
**53**	−33.34	1	Ser373	Phe338, Phe374, Val367, Cys336, Leu368, Ser373
**54**	−35.10	0	0	Ser371, Ser373, Phe338, Leu335, Cys336
**55**	−29.06	1	Cys336	Cys336, Phe342, Val367, Leu335, Ser371, Asn343
**56**	−34.90	2	Ser371, Cys336	Phe374, Phe338, Ser371, Val367, Cys336, Leu368, Ser373
**57**	−34.77	0	0	Ser373, Phe338, Phe342, Cys336, Gly339
**58**	−30.22	1	Ser371	Phe342, Asn343, Phe374, Ser371, Leu368
**59**	−34.70	1	Ser371	Ser373, Asn343, Phe374, Ser371, Leu368, Val367, Leu335
**NAG**	−21.39	1	Ser371.	Phe374, Phe342, Phe338

**Table 4:** Free binding energies of studied isoflavonoids and ligand to coronavirus M^pro^ and amino acid residues involved in H. bonds and hydrophobic interaction.

Comp.	Binding Energy (kcal mol^−1^)	No. of H. Bonds	Involved Amino Acid Residues	Amino Acid Residues Involved in Hydrophobic Interaction
**1**	−37.38	1	Glu166	Phe140, Leu141, Gln189, His41, Tyr54, Glu166
**2**	−35.91	1	Phe140	Phe140, Leu141, Gln189, His41, Tyr54, Glu166
**3**	−36.08	1	Glu166	Phe140, Leu141, Gln189, His41, Tyr54, Glu166
**4**	−37.99	1	Glu166	Phe140, Leu141, Gln189, His41, Tyr54, Glu166
**5**	−38.45	2	Thr190, Leu141	Phe140, Leu141, Gln189, His41, Tyr54, Glu166
**6**	−41.41	1	Glu166	Phe140, Leu141, Asn142, His163, Tyr54, Glu166
**7**	−40.11	1	Glu166	Phe140, His172, Glu166, His163, His164, Gln189
**8**	−42.73	3	Glu166, Cys145, His163	Phe140, Leu141, Glu166, His163, His164, Gln189
**9**	−33.98	1	Phe140.	Phe140, Leu141, Gln189, His41, Tyr54, Glu166
**10**	−35.25	2	Glu166, Phe140.	Phe140, Leu141, Gln189, His41, Tyr54, Glu166
**11**	−32.19	1	Glu 166	Phe140, Leu141, Gln189, His41, Tyr54, Glu166
**12**	−41.55	3	Gln192, His41, Arg188	Glu166, Met 165, Gln192, His41, His164, His172
**13**	−40.51	1	Glu 166	Met 165, Cys145, His41, Asn142, Glu166
**14**	−39.89	1	Glu 166	His163, Met 165, Cys145, His41, Glu189, Glu166
**15**	−37.34	1	Glu166	Phe140, Met 165, Asp187, His41, Glu189, Glu166
**16**	−39.05	6	Glu166, Cys145, Thr26	Glu166, Cys145, Thr26, His41, Met 165, Glu189, Leu27
**17**	−40.60	1	Gly143	Glu166, Cys145, Thr26, His4, Met 165, Gln189, Gln192
**18**	−35.58	0	0	Glu166, Phe140, Gly143, Asp187, Met 165, Gln189
**19**	−37.26	0	0	Glu166, Phe140, Cys145, Asp142, Met 165, Gln189
**20**	−34.97	1	Glu 166	Phe140, Gln189, His41, Ser144, Tyr54, Glu166
**21**	−38.42	1	Phe140	Phe140, Leu141, Gln189, His41, Met165, Leu140, Glu166
**22**	−40.14	1	Phe140	Phe140, Leu141, Gln189, His164, Met165, Leu140, Glu166
**23**	−40.24	0	0	Glu166, Phe140, Leu141, Gln189, His41, Met165, Leu140
**24**	−38.90	2	Phe140, Glu166	Glu166, Phe140, Leu141, Gln189, His164, Met165, Leu140, Cys145
**25**	−34.43	2	Phe140, Glu166	Glu166, Phe140, His41, Gln189, His164, Met165, Cys145
**26**	−36.39	1	Phe140	Glu166, Phe140, His41, Gln189, His164, Met165
**27**	−38.58	3	Gly143, Cys145, Thr26	Glu166, Gly143, Gln189, Cys145, Thr26, Met165
**28**	−47.62	1	His41	Glu166, Phe140, His41, Gln189, His164, Met165, Cys145, Leu141
**29**	−49.64	3	Glu166, Tyr54, Asp187	Phe140, Gln189, His172, Met165, Tyr54, Asp187, Leu167, Glu166
**30**	−48.39	3	Glu166, Tyr54, Asp187	His41, Gln189, His163, Met165, Tyr54, Asp187, Leu167, Glu166
**31**	−48.32	1	Glu 166	Phe140, Gln189, His41, Met165, Tyr54, Glu166
**32**	−38.31	1	Cys145	Gln189, His41, Met165, Cys145, Glu166
**33**	−43.52	2	Gln189, Gly143	Met165, Gln189, Gly143, Glu166
**34**	−45.48	2	Glu166, Cys145	Glu166, Phe140, His41, Gln189, His164, Met165, Cys145
**35**	−41.38	1	Gly143	Glu166, Gly143, Leu107, Gln192, His164, Met165, Cys145
**36**	−42.29	4	His164, Cys145, Ser144, Leu141	Gln189, His172, Met165, Glu166 His164, Cys145, Ser144, Leu141
**37**	−48.13	4	Met165, Thr190, His41, Cys145	Glu166, Met165, Thr190, His41, Cys145, Gln189
**38**	−43.30	1	Glu 166	Gln189, His163, Met165, Ser144, Glu166, Leu167
**39**	−38.05	4	Glu166, Cys145	Glu166, Cys145, Met165, Asn142
**40**	−36.12	2	Glu166, His163	Glu166, His163, Phe140, Met165
**41**	−38.22	3	Gln189, Asp187, Tyr54	Gln189, Met165, His163, Glu166
**42**	−37.17	1	Glu166	Glu166, Leu141, Gln189, Gly143
**43**	−35.41	1	Asp187	Glu166, Leu141, Met165, Ser144
**44**	−36.62	1	Glu166	Gln189, Met165, His172, Glu166, His163
**45**	−40.48	4	Ser144, Cys145, Thr26, Gly143	Cys145, Thr26, His163, Met165, Asn142
**46**	−40.84	1	His163	Glu166, His163, Phe140, Met165
**47**	−35.39	1	Glu166	Glu166, Asn142, His164, Met165
**48**	−40.40	1	His163	Glu166, Leu141, Met165, Gln189
**49**	−43.83	4	Glu166, Cys145, His41	Glu166, Cys145, His41, Met165, Asn142, Leu141
**50**	−43.91	1	Glu166	Glu166, Leu141, Met165, Gln189
**51**	−46.15	2	Glu166	Glu166, Ser144, Gln189, His41
**52**	−41.20	1	Glu166	Glu166, Leu141, Met165, Gln189, Asn142
**53**	−46.90	2	Glu166, Phe140	Glu166, Gln189, Leu141, Met165, His172, Phe140
**54**	−50.79	1	Glu166	Glu166, Gln189, Leu141, Met165, His172
**55**	−40.56	1	Thr26	Asn142, Glu166, Asn142, Leu141
**56**	−48.29	3	Glu166, His41	Glu166, His41, Met165, Asn142, His164
**57**	−49.89	2	Gly143, Arg188	Glu166, Gln189, Leu141, Met165, His163
**58**	−42.63	2	Glu166, Leu141	Glu166, Gln189, Leu141, Met165, His172
**59**	−48.11	2	Gly143, Leu141	Glu166, Gln189, Met165,
**N3** **(Co-crystallized ligand)**	−62.84	4	Gln189, Tyr54, Asp142, Asp187.	Phe140, Glu166, His172, Thr190, Gln189, Tyr54, Asp142, Asp187.

**Table 5:** Toxicity properties of isoflavonoids (**1**–**59**) and semiprever.

Comp.	FDA Rodent Carcinogenicity	Carcinogenic Potency TD_50_ (Rat) ^a^	Rat MTD (Feed) ^b^	Rat Oral LD_50_ ^c^	Rat Chronic LOAEL ^d^	Ocular Irritancy	Skin Irritancy
**1**	Non-Carcinogen	60.47	0.516	1.40	0.107	Irritant	None
**2**	Non-Carcinogen	67.14	0.334	1.41	0.089	Irritant	None
**3**	Non-Carcinogen	10.43	0.225	0.81	0.068	Irritant	None
**4**	Non-Carcinogen	5.69	0.231	0.17	0.072	Irritant	None
**5**	Non-Carcinogen	5.73	0.234	0.17	0.071	Irritant	None
**6**	Carcinogen	35.33	0.239	0.77	0.024	Irritant	None
**7**	Non-Carcinogen	6.23	0.096	0.48	0.019	Irritant	None
**8**	Non-Carcinogen	33.45	0.529	1.06	0.074	Irritant	Mild
**9**	Carcinogen	4.43	0.122	0.14	0.027	Irritant	Mild
**10**	Carcinogen	28.52	0.126	0.16	0.011	Irritant	None
**11**	Non-Carcinogen	7.51	0.192	0.55	0.015	Irritant	None
**12**	Non-Carcinogen	193.96	0.078	0.10	0.004	Mild	None
**13**	Non-Carcinogen	5.27	0.255	1.07	0.865	Mild	None
**14**	Non-Carcinogen	9.10	0.164	1.13	0.325	Mild	None
**15**	Non-Carcinogen	7.32	0.288	2.03	0.147	Mild	None
**16**	Non-Carcinogen	7.91	0.230	1.67	0.155	Mild	None
**17**	Non-Carcinogen	8.98	0.184	1.18	0.152	None	None
**18**	Non-Carcinogen	8.40	0.205	1.69	0.309	Mild	None
**19**	Non-Carcinogen	0.77	0.069	0.39	0.130	None	Mild
**20**	Non-Carcinogen	0.59	0.061	0.20	0.145	None	Mild
**21**	Non-Carcinogen	6.40	0.181	1.44	0.229	Mild	None
**22**	Non-Carcinogen	5.73	0.205	2.36	0.390	Mild	None
**23**	Non-Carcinogen	0.44	0.077	0.42	0.282	Mild	Mild
**24**	Non-Carcinogen	6.88	0.288	4.66	0.863	Mild	None
**25**	Non-Carcinogen	19.50	0.181	0.97	0.191	None	None
**26**	Non-Carcinogen	10.75	0.145	1.01	0.281	Mild	None
**27**	Non-Carcinogen	29.81	0.184	1.74	0.054	Mild	None
**28**	Non-Carcinogen	19.03	0.199	0.77	0.035	None	None
**29**	Non-Carcinogen	25.03	0.080	0.35	0.055	Severe	None
**30**	Non-Carcinogen	2.33	0.097	0.45	0.039	Severe	None
**31**	Non-Carcinogen	2.33	0.097	0.45	0.039	Severe	None
**32**	Non-Carcinogen	20.46	0.128	0.26	0.074	Mild	None
**33**	Non-Carcinogen	73.66	0.197	0.29	0.013	Mild	Mild
**34**	Non-Carcinogen	25.44	0.526	0.92	0.018	Severe	None
**35**	Non-Carcinogen	6.87	0.236	0.37	0.013	Mild	None
**36**	Non-Carcinogen	322.42	0.764	0.84	0.029	Severe	None
**37**	Non-Carcinogen	165.35	0.303	1.39	0.008	Mild	None
**38**	Non-Carcinogen	19.21	0.153	0.46	0.024	Mild	None
**39**	Non-Carcinogen	35.43	0.576	0.70	0.012	Severe	None
**40**	Non-Carcinogen	4.926	0.216	0.44	0.015	Mild	None
**41**	Non-Carcinogen	6.31	0.381	0.98	0.075	Severe	None
**42**	Non-Carcinogen	5.95	0.428	0.71	0.026	Mild	None
**43**	Non-Carcinogen	6.31	0.381	0.91	0.044	Mild	None
**44**	Non-Carcinogen	5.81	0.475	1.12	0.041	Mild	None
**45**	Non-Carcinogen	5.28	0.402	0.76	0.037	Mild	None
**46**	Non-Carcinogen	3.25	0.668	1.10	0.174	Mild	None
**47**	Non-Carcinogen	4.02	0.395	0.65	0.084	None	None
**48**	Non-Carcinogen	126.90	0.545	0.39	0.009	Severe	None
**49**	Non-Carcinogen	14.44	0.284	0.32	0.024	Mild	None
**50**	Non-Carcinogen	14.44	0.284	0.20	0.008	Mild	None
**51**	Non-Carcinogen	16.34	0.226	0.14	0.008	Mild	None
**52**	Non-Carcinogen	21.43	0.226	0.46	0.010	Mild	None
**53**	Non-Carcinogen	18.79	0.150	0.34	0.008	Mild	None
**54**	Non-Carcinogen	18.79	0.150	0.26	0.007	Mild	None
**55**	Non-Carcinogen	14.61	0.226	0.32	0.053	Severe	None
**56**	Non-Carcinogen	116.75	0.562	0.42	0.006	Severe	None
**57**	Non-Carcinogen	177.62	0.291	0.36	0.004	Severe	None
**58**	Non-Carcinogen	5.28	0.156	0.18	0.016	Mild	None
**59**	Non-Carcinogen	15.21	0.208	0.35	0.014	Mild	None
**Simeprevir**	Non-Carcinogen	0.28	0.003	0.21	0.002	Irritant	None

^a^ TD _50_, tumorigenic dose rate 50, Unit: mg kg^−1^ body weight/day; ^b^ MTD, maximum tolerated dose, Unit: g kg^−1^ body weight; ^c^ LD_50_, median lethal dose, Unit: g kg^−1^ body weight; ^d^ LOAEL, lowest observed adverse effect level, Unit: g kg^−1^ body weight.

## Data Availability

Not applicable.

## Sample Availability

Samples of the compounds are not available from the authors.
